# Treating bacterial infections with bacteriophages in the 21st century

**DOI:** 10.4102/sajid.v37i1.346

**Published:** 2022-03-29

**Authors:** Christoffel J. Opperman, Justyna M. Wojno, Adrian J. Brink

**Affiliations:** 1 National Health Laboratory Service, Green Point Laboratory, Cape Town, South Africa; 2 Department of Pathology, Faculty of Health Science, University of Cape Town, Cape Town, South Africa; 3 Lancet Laboratories, Cape Town, South Africa; 4 Microbiology Laboratory, National Health Laboratory Service, Groote Schuur Hospital, Cape Town, South Africa

**Keywords:** bacteriophage, bacteriophage therapy, phage, non-lytic phage, vaccination, gene-transfer, endolysins, enzybiotics, artilysins

## Abstract

Bacteriophages (phages) were discovered in the early part of the 20th century, and their ability to eliminate bacterial infections as bacterial viruses gathered interest almost immediately. Bacteriophage therapy was halted in the Western world due to inconclusive results in early experiments and the concurrent discovery of antibiotics. The spread of antibiotic-resistant bacteria has elicited renewed interest in bacteriophages as a natural alternative to conventional antibiotic therapy. Interest in the application of bacteriophages has also expanded to include the environment, such as wastewater treatment, agriculture and aquaculture. Although the complete phage is important in bacteriophage therapy, the focus is shifting to purified phage enzymes. These enzymes are an attractive option for pharmaceutical companies with their patent potential. They can be bio-engineered for enhanced adjuvant properties, such as a broadened spectrum of activity or binding capability. Enzymes also eliminate the concern that the prophage might integrate resistance genes into the bacterial genome. From a clinical perspective, the first randomised clinical controlled phage therapy trial was conducted with more pioneering phase I/II clinical studies on the horizon. In this opinion paper, the authors outline bacteriophages as naturally occurring bactericidal entities, their therapeutic potential against antibiotic-resistant bacteria and compare them to antibiotics. Their potential multipurpose application in the medical field is also addressed, including the use of bacteriophages for vaccination, and utilisation of the antimicrobial enzymes that they produce.

## Introduction

As anticipated by Alexander Fleming, soon after discovering penicillin in the 1920s, 14% of *Staphylococci* isolated from patients in a London hospital demonstrated resistance to penicillin by 1946.^1^ Antibiotic resistance has subsequently proliferated at an alarming rate to the level that by 2050, 10 million deaths due to antibiotic-resistant infections may occur.^2^ The emergence and spread of pan-drug resistant bacteria render the need for new treatment approaches a priority.

**FIGURE 1:**
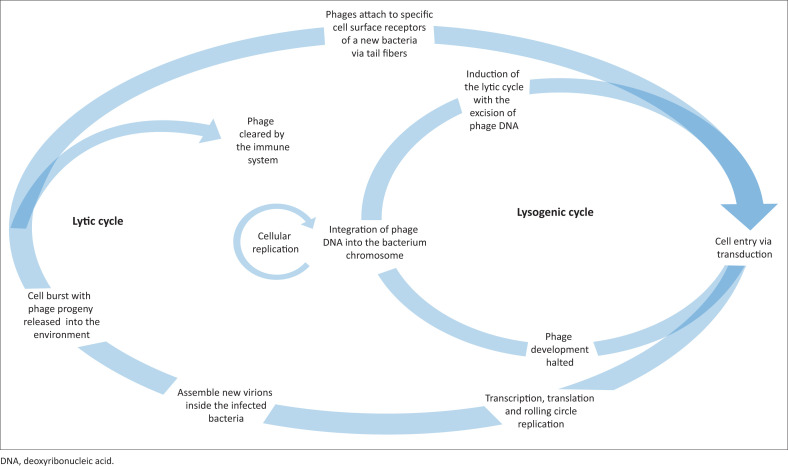
Life cycle of bacteriophages. Adsorption occurs between the bacteriophage (phage) and bacterium cell surface receptors. The phage protein capsule is left behind once the linear DNA enters the infected bacterium to form circular deoxyribonucleic acid molecules. From here, two life cycles can be entered. During the lytic cycle (bacteriophage therapy), tail fibres and protein capsids (heads) are formed during transcription and translation using the biosynthetic apparatus of the bacteria, such as ribosomes. The phage particles are assembled, and the viral genome is packaged within the capsids. Holin proteins and endolysins are involved in cell lysis and phage progeny release. The second pathway is termed as the lysogenic cycle. On this path, the temperate phage DNA is integrated into the bacterium chromosome (prophage), and phage development is halted. Excision of phage DNA from the chromosome can allow the phage to re-enter the lytic life cycle.

Bacteriophages are viruses that kill bacteria following amplification and bacterial lysis to release virion progeny into the environment (lytic-lifecycle). Bacteriophage therapy exploits this lifecycle (Figure 1) without the integration of the bacteriophage (prophage) into the host genome (lysogenic lifecycle).^3^ Alternative approaches include using non-lytic bacteriophages as a delivery vehicle to transport deoxyribonucleic acid (DNA) vaccines or using purified phage components such as cell wall hydrolases as novel modalities to treat antibiotic-resistant infections. Bacteriophage therapy does have potential limitations that include but are not limited to toxic shock following bacterial lysis or the generation of neutralising bacteriophage antibodies after repeated treatment.^4^

## Comparison: Bacteriophages versus antibiotics

Lytic bacteriophages offer several potential advantages over antibiotic therapy (Table 1). Most importantly, unlike antibiotics, they are pathogen-specific, thus reducing the extensive disruption of microbial flora due to high specificity. Maura et al., in 2011, constructed a stable mouse intestine model to study the possible long-term effect of self-replicating bacteriophages within a mammalian poly-microbial niche. The study concluded that no change in the faecal bacteria levels was observed.^5^

**TABLE 1:** A comparison in the therapeutic use of bacteriophage and antibiotics.

Characteristic	Bacteriophage	Antibiotics	Reference
1. Side effects	Only a few minor side effects have been reported, mainly because of endotoxins released from bacteria following therapeutic phage therapy *in vivo*.	Multiple side effects can complicate and alter antibiotic application. These include intestinal disorders, allergies and the proliferation of secondary infections such as yeasts.	9
2. Specificity	Bacteriophages are highly specific. In principle, they only affect the targeted bacterial species. Therefore, secondary infections are usually avoided with minimal impact on health protecting flora. In addition, phage resistance is limited due to a narrow host range.	Antibiotics target both normal micro-flora and pathogenic bacteria. Secondary severe infections may develop because of a microbial imbalance. In addition, a significant number of bacteria are exposed to and acquire antibiotic resistance.	19
3. Dose	They are available where they are most needed because they self-replicate at the site of the infection and achieve efficacy after a single dose (Auto-dosing).	Antibiotic concentrations are affected by multiple factors that impact on availability at the site of the infection.	6
4. Selection	Phage selection is a swift process that can be accomplished in weeks or days (against bacteriophage-resistant bacteria). The concept of phage biobanks is increasing.	It is time-consuming to develop a novel antibiotic and may take several years (against antibiotic-resistant bacteria).	58, 68
5. Immune response	Bacteriophages can elicit an immune response.	Antibiotics are small molecules that do not generate an immune response as a rule.	7
6. Resistance	Bacteria that acquire resistance to a phage remain vulnerable to other bacteriophages with a comparable target range. Also, whole-genome sequencing paves the way to predict positive phage-bacterial interactions.	Antibiotic resistance is not restricted to the targeted pathogen.	58, 69
7. Cross-resistance	Bacteriophages alter their structure naturally to combat host resistance. Specific antibiotic-resistant mechanisms do not affect bacteriophage-resistance.	Antibiotics cannot alter their structure as a non-living entity. Therefore, exposure to antibiotics can readily translate into (multi)drug resistance. Furthermore, cross-resistance is not uncommon.	70
8. Biofilm penetration	Specific phages hold the potential to clear biofilms effectively. This is achieved by displaying biofilm exopolymer-degrading depolymerases or lysing one bacterial layer at a time.	Most antibiotics do not penetrate biofilms.	69
9. Killing capability	Bactericidal in nature, causing cell rupture after an obligatory lytic phage successfully infected a bacterium. The pathogen loses its viability. Phage progeny released into the environment, infecting remaining bacteria.	Not all antibiotics are bactericidal.	65
10. Manufacturing	Bacteriophages occur naturally. In addition, phage discovery and isolation are relatively cheap.	Antibiotics are primarily laboratory-synthesised molecules engineered to combat pathogens. It is time-consuming and expensive to develop a novel antibiotic effective against multidrug-resistant organisms.	68

Another major advantage of using therapeutic phages is their self-limiting amplification capability, as they only persist as long as the bacteria are present. Notably, the use of localised phage therapy leads to deep penetration as long as the infection is present, as opposed to antibiotics, where from a pharmacokinetic point of view specific targets need to be maintained with repetitive doses to maintain concentrations.^6^ As auto-replication occurs after one dose, less phage is required to achieve the optimum therapeutic effect.

Most bacteriophages promote a strong antibody response and are highly immunogenic.^7^ It is unclear if this response is because of the phage itself or the lysed bacterial components. Zimecki et al. demonstrated that prophylactic phage administration in an immunosuppressed mouse model could overcome neutrophil deficit in the clearance of *Staphylococcus aureus* infection and mobilise cells from both lymphocytic as well as myelocytic lineages. It was postulated in this study that phages could promote the recruitment of immature neutrophil cell types from bone marrow precursors and trigger a rapid output of functional mature neutrophils into the peripheral circulation to clear the bacterial infection.^8^

Endotoxin released from bacteria lysed *in vivo* may lead to potential health concerns in patients. A particularly useful strategy may be to use lysis deficient bacteriophages, which decreases the release of pro-inflammatory cytokines.^9^ However, considering the minor side effects reported for bacteriophage therapy, such as occasional serum-like illness after intravenous administration, multiple side effects have been observed with antibiotics. Anaphylaxis following bacteriophage therapy has never been described in the literature. The initial reporting of ‘shock’ was most likely because of a low level of purification and contaminants in phage preparations.^10^ In this regard, protein toxins produced by pathogenic species and gram-negative bacteria endotoxins are impurities in phage lysates that are harmful to humans. Therefore, similar to antibiotic purification, procedures should be rapid, scalable and efficient in removing impurities to ensure patient safety.^11^

Bacteriophage therapy is a promising alternative to antibiotics in vulnerable population groups such as pregnant population and neonates, where numerous antibiotic contraindications pertain. Minimal disruption of the host microbiota during the antenatal and perinatal periods is beneficial. In addition, the transplacental transfer of phages has been observed in guinea pigs following intravenous maternal phage administration. Further studies are required to explore in utero bacteriophage treatment strategies.^12^

## Phage-antibiotic combination therapy

There is a clear potential to use phage therapy concurrently with antibiotics, especially with increasingly antibiotic-resistant pathogens.^13^ This is especially true when the phage component is applied at high titres topically and therefore not easily removed by the immune system. Furthermore, phages encapsulated in hydrogels can result in a long-term release of active particles, whilst liposome preparations applied topically protect against immune system components and enzymatic hydrolysis.^14,15^ It is unlikely; however that bacteriophage therapy will replace antibiotics but combining antibiotics with phage therapy should reduce bacterial resistance because the pathogen must acquire two separate mechanisms to survive. Altering binding targets for both antibiotics and phages may come at a fitness cost for the bacteria because mutations can influence essential biological functions. The concept of a trade-off of antibiotic resistance mechanisms and phage resistance is referred to as ‘phage steering’.^16,17^ Further research is required to explore the evolutionary response of bacterial strains to the phage-antibiotic combinations. The argument supports the idea that a phage can be selected against every antibiotic or phage-resistant bacterium from an evolutionary standpoint. The ongoing process of natural selection achieves this.^18^

A strategy to prevent phage resistance would be to use complex mixtures of bacteriophages or ‘phage cocktails’ to circumvent phage resistance.^19^ Alternatively, the non-lytic bacteriophage discussed in the following sections may be used to deliver DNA encoding phage-proteins that affect bacterial cell division, DNA replication, transcription and translation.^20^

## Bacteriophage mediated gene and antimicrobial agent transfer

Many novel antibiotic agents are excluded from clinical use because of their inability to be selected explicitly for bacterial versus human cells. This may result in toxicity. During ‘phage display’, an antigen gene is fused with a gene coding for a coat-surface protein and, when expressed, it is displayed as a fusion protein on the capsule of the bacteriophage.^21^ In targeted drug delivery, a targeting moiety is displayed on the filamentous phage coat with phage display. The cytotoxic drug is then chemically linked with an ester bond to the bacteriophage. The drug is a prodrug with no cytotoxic activity in the conjugated state. Once the prodrug is dissociated from the phage at the target site in a controlled manner, it is activated. Targeted drug delivery has been shown *in vitro* between *S. aureus* and chloramphenicol.^22^ Adapted bacteriophage vectors have been used to restore antibiotic sensitivity by reversing antibiotic resistance in pathogens. For example, a proof-of-concept model exploited the temperate phage to introduce the *rpsL* and *gyrA* genes and confer sensitivity to streptomycin and nalidixic acid, respectively, where these antibiotics were resistant.^22,23^ Unlike conventional bacteriophage therapy, the authors proposed a scenario where these phages can deliver genetic constructs and render bacteria sensitive to previously resistant antibiotics. Rather than administering phages to the patient, they may also be applied to hospital surfaces and, in the process, potentially reduce environmental contamination by antibiotic-resistant pathogens.

Various methods have been employed to increase phage uptake and stability to target intracellular pathogens. In this Trojan horse approach, pages are loaded onto a carrier vehicle during receptor-mediated uptake. These novel technologies include nanocrystal, liposome, polymer encapsulation and delivery, genetically engineered phage, nanofibre entrapment, nanoparticle absorbed and hydrogel embedded phages.^24^

## Bacteriophage vaccination

Bacteriophages vaccination is an innovative and novel vaccine system.^25,26^ This is a bio-engineered structure that entails a DNA vaccine contained within a phage capsule under the expression of a eukaryotic promoter.^21^ Once the phage transporting the DNA vaccine is engulfed into a eukaryotic antigen-presenting cell, the vaccine gene is expressed. Following this, major histocompatibility complexes (MHC) present the processed antigens to the immune system. Antigens presented to MHC II generate an antibody response, while a cellular response is activated through cross-priming with the MHC I pathway. Phage display vaccines can also directly activate B cells to produce antibodies.^26^ Haq et al. reported on studies proposing a hybrid phage with phage display and DNA vaccine capability.^27^ The advantage of an engineered DNA phage vaccine is to treat and prevent infection. Bacteriophage vaccines are also suitable to immunise large communities because they are relatively easy to apply and inexpensive to produce. Jepson and March, in 2004 postulated the concept of bacteriophage DNA vaccines administered orally via drinking water.^28^ Notably, during such a proposed vaccination programme, a high phage titre would be required as it appears that gut transit and phage penetration after oral administration are dose-dependent.^29^

## Bacteriophage enzymes

Purified lysins are promising antibacterial phage components currently under development. They consist of a class of cell wall hydrolases that translocate into the peptidoglycan wall of bacteria and cleave the bonds, which results in the release of viral progeny during a viral infection cycle. Endolysins have a characteristic structure with multiple cell wall binding domains. They degrade the peptidoglycan wall with lytic transglycosylase, amidase glycosidase or endopeptidase activities.^30^ Their synergistic mechanism of action with a range of other antimicrobials or fellow peptidoglycan hydrolases is of interest. Endolysins are less likely to invoke resistance because of the coevolution of bacteriophages and bacteria.^31^ The purified lysins can also be applied directly to sensitive bacteria. Enzyme preparations include aerosols for inhalation, formulations for systematic injection, topical ointments and creams.^32^ The lytic activity spectrum of lysins is usually narrow because lysin receptors are distributed in a strain-specific manner. Furthermore, the binding affinity is equivalent to mature antibodies, making them an irreversible inhibitor.^33^ This alone promises that lysins could be highly efficacious when compared to antibiotics. An anti-staphylococcal lysin (exebacase or formally known as CF-301) demonstrated a 42.8% higher clinical response rate when given alongside the standard of care antibiotic compared to the antibiotic alone when treating methicillin-resistant *Staphylococcus aureus* (MRSA) bacteremia, including infective endocarditis. As a result, this is the first lysin to have entered a phase III human trial to treat *S. aureus*.^34,35,36^

An interesting feature of these ‘enzybiotics’ is their structural versatility.^37^ An enzybiotic is a bacteriophage enzyme with cell wall degrading capability against bacteria or fungi. It is a hybrid term derived from the words enzyme and antibiotic. The cell wall hydrolysis and receptor binding domains of the structure can be separated while maintaining their activity. These domains can be fused in a complementary fashion to other lysins to alter catalytic function, redirect binding or both. These bioengineered lysins are the so-called next-generation lysins.^38^ Artilysins are a group of next-generation lysins with an additional lipopolysaccharide disrupter to increase outer membrane penetration of gram-negative bacteria. A limited number of lysins can be recombined to generate a highly variable number to avoid bacterial resistance. So far, no neutralising antibodies or lysine resistance has been detected under the same conditions that favour antibiotic resistance and similarly no toxic side effects have been demonstrated.^39^ Lysins have shown promising results to clear pathogens on the mucous membranes in animal models and *in vitro*. The chimeric ectolysin P128 was manufactured to clear nasal MRSA infections. *In situ* efficacy was seen with a P128 hydrogel formulation against *Staphylococci* recovered from the nares of 31 healthy individuals, including mupirocin-resistant isolates. In addition, an MRSA nasal decolonisation rat model compared P128 formulated as a hydrogel to 2% mupirocin ointment. P128 cleared colonisation twofold, whereas the mupirocin alone was ineffective in the rat nares.^37,40,41^ A randomised, double-blind placebo-controlled study to determine the efficacy and safety of P128 applied intra-nasally to healthy volunteers has been completed (results not publicly available) to evaluate *S. aureus* clearance and re-colonisation rates.^42^

Although further attention is needed in preclinical testing with lysins, applications could include the treatment of antibiotic-resistant bacterial biofilms on medical devices such as catheters.^43^ LysGH15 inhibits biofilm formation *in vitro*, but at higher doses, it distorts the integrity of the biofilm by dislodging the bacteria instead of destroying the biofilm matrix.^44^ The bacteriophage-derived lysin PlySs2 reduced colony-forming units by 99% and biofilm by 75% relative to vancomycin *in vitro*.^45^ Lastly, killing slow or non-growing organisms associated with bacterial infections of a chronic nature that may be naturally resistant to phage treatment or antibiotics could also be susceptible to lysins.^39,46^

## Clinical case studies and trials using bacteriophages

Several case reports highlight the potential use of phages in various clinical syndromes. Case studies illustrating the successful eradication of multidrug-resistant (MDR) *Pseudomonas aeruginosa* in ventilated patients and *Klebsiella pneumoniae* lung infections are promising.^47,48^ Furthermore, phage therapy co-administered with antibiotics has been successfully utilised in a refractory case of a complicated intra-abdominal infection because of MDR *Acinetobacter baumannii.*^49^ Even though phage resistance emerged in this patient with a pancreatic pseudocyst infection, it was rapidly circumvented with the selection and purification of a salvage phage that was subsequently added to the treatment regime.^49^ Notably, a case series in critically ill patients with prosthetic or native valve endocarditis because of *S. aureus* treated with a bacteriophage displayed reduced bacterial counts, clinical improvement and corresponding decreases in inflammatory markers.^50^ Other applications include gut decolonisation strategies, such as eradicating MDR, carbapenemase-producing *K. pneumoniae* isolates, following intra-rectal and oral therapy with a lytic bacteriophage preparation.^51^

Although several phase I/II clinical studies on bacteriophage therapy have been published with many case reports that include different routes of phage administration, sources of infection and targeted pathogens,^52^ only two have progressed to phase III to our knowledge. The first is an active open-label, single-group assigned intervention of a nebulised liquid pyobacteriophage complex that entails the irrigation of mucous membranes in children with confirmed acute tonsillitis.^53^ A phase III randomised, placebo-controlled, double-blind trial was recently conducted in patients undergoing transurethral resection of the prostate with urinary tract infections. Treatment with intravesicular phage administration was safe and non-inferior in outcome compared to standard antibiotic treatment.^54,55^

The poor clinical efficacy of phage therapy in many previous phase I/II trials could be related to design and technical issues.^56^ Possible factors for phage failure include sub-optimal titres and quality of phage preparations, the production of serum anti-phage antibodies, insufficient phage coverage of targeted pathogens, phage-eukaryotic cell interactions that might modify the effect of phage therapy and external factors such as medication (proton pump inhibitors, antibiotics) as well as food and drinks (yoghurt, alcohol).^57,58^ A better focus and understanding of these confounding factors during trial design might lead to more favourable outcomes in the future.

## Bacteriophages for laboratory identification of bacteria

Although not the focus of this overview, mention should be made to bacteriophage’s unique capability to detect specific bacteria for pathogen diagnostic purposes. Fluorescently stained phages that bind to targeted bacteria can be visualised under a fluorescence microscope as halo structures. Alternatively, genetically engineered phages express reporter genes during phage-bacteria binding. Quantum dots (nanocrystals) bioconjugated to the capsid surface can be visualised using a microscope during phage-bacteria interaction. In optical and electrochemical transducer systems, phage heads are immobilised onto electrodes exposing their tails to capture specific bacteria that can identify microorganisms.^59^

## Conclusion and future direction

Phage research is gaining interest on the African continent, but few groups have entered phage product design and development stages. As a result, *in vivo* studies, phage safety, efficacy and phage biobanking in the African context remain a priority.^60^ Although no single phage manufacturing centre has been established in Africa yet, research groups have been initiated to foster collaboration with Phages for Global Health.^60^ This initiative is focused on initially teaching scientists in African countries to isolate and characterise phages.

It remains unclear if phages should be classified as biological or chemical agents (lysins), food additives or pharmaceutical drugs. This uncertainty complicates legislation, regulatory processes for bacteriophage therapy and investment.^60^ Current regulatory frameworks should support phage research and academic institutions to promote development, be flexible in compassionate use cases and be open to possible alternative pathways for the approval of phage therapy.

In this regard, a pertinent question for the future of phage therapy is whether manufacturers are willing to invest in the development of a product with limited patent protection. Intellectual property status is one of the major contributing factors to the general reluctance to develop phage therapy in developed countries. Pharmaceutical companies will not undergo expensive and rigorous clinical trials for drug approval and registration without patent protection.^61,62^ The possibility for an individual phage to be patented is unlikely; however, firms have adopted strategies to commercialise phage therapy. This includes patenting specific phage sequences or phage components mentioned above. Establishing regional phage banks across Africa that have already isolated, characterised, sequenced and purified phages could contribute to sustainable and cost-effective phage therapy.^60,63^ In addition, inaugurating networks of phage banks across the globe between reference phage laboratories and low-income regions could further help transfer knowledge and lower the cost of bacteriophage therapy.

In the future, treatment of antibiotic-resistant bacterial infections with phage therapy might be preceded by bacteriophage control of pathogens from an ecological perspective. To prevent infection with phage rather than to treat it is the focus for future research in agriculture, water treatment and food-related industries.^64,65,66^ Until bacteriophage bio-control becomes the next frontier against pathogens, focusing on research that could provide regulatory bodies with the evidence to justify phage commercialisation and clinical use remains essential. This includes research on their complimentary bactericidal activity against antibiotic-resistant infections and their ability to reduce the gastro-intestinal carriage of MDR pathogens, from an infection prevention control point of view. In addition, designing phage formulations specifically in the African context that are easy to mass administer, have a long shelf life, are robust for transportation at different temperatures, and obtaining large-scale production skills should not be ignored.^67^ Finally, clinical data on bacteriophage and phage-derived products remain limited, with more rigorous clinical trials and reference standards needed to substantiate phage efficacy, determine appropriate treatment regimens and confirm its safety profile.
